# Inhibitors of Deubiquitinating Enzymes Block HIV-1 Replication and Augment the Presentation of Gag-Derived MHC-I Epitopes

**DOI:** 10.3390/v9080222

**Published:** 2017-08-12

**Authors:** Christian Setz, Melanie Friedrich, Pia Rauch, Kirsten Fraedrich, Alina Matthaei, Maximilian Traxdorf, Ulrich Schubert

**Affiliations:** 1 Institute of Virology, Friedrich-Alexander University Erlangen-Nürnberg (FAU), Erlangen 91054, Germany; Christian.Setz@viro.med.uni-erlangen.de (C.S.); Melanie.Friedrich@viro.med.uni-erlangen.de (M.F.); Pia.Rauch@viro.med.uni-erlangen.de (P.R.); Kirsten.Fraedrich@viro.med.uni-erlangen.de (K.F.); Alina.Matthaei@viro.med.uni-erlangen.de (A.M.); 2 Department of Otorhinolaryngology, Head and Neck Surgery, Friedrich-Alexander University Erlangen-Nürnberg (FAU), Erlangen 91054, Germany; maximilian.traxdorf@uk-erlangen.de

**Keywords:** HIV-1, DUB-inhibitors, UPS, DUBs, ubiquitination, MHC-I, proteasome inhibitors

## Abstract

In recent years it has been well established that two major constituent parts of the ubiquitin proteasome system (UPS)—the proteasome holoenzymes and a number of ubiquitin ligases—play a crucial role, not only in virus replication but also in the regulation of the immunogenicity of human immunodeficiency virus type 1 (HIV-1). However, the role in HIV-1 replication of the third major component, the deubiquitinating enzymes (DUBs), has remained largely unknown. In this study, we show that the DUB-inhibitors (DIs) P22077 and PR-619, specific for the DUBs USP7 and USP47, impair Gag processing and thereby reduce the infectivity of released virions without affecting viral protease activity. Furthermore, the replication capacity of X4- and R5-tropic HIV-1_NL4-3_ in human lymphatic tissue is decreased upon treatment with these inhibitors without affecting cell viability. Most strikingly, combinatory treatment with DIs and proteasome inhibitors synergistically blocks virus replication at concentrations where mono-treatment was ineffective, indicating that DIs can boost the therapeutic effect of proteasome inhibitors. In addition, P22077 and PR-619 increase the polyubiquitination of Gag and thus its entry into the UPS and the major histocompatibility complex (MHC)-I pathway. In summary, our data point towards a model in which specific inhibitors of DUBs not only interfere with virus spread but also increase the immune recognition of HIV-1 expressing cells.

## 1. Introduction

The homeostasis of eukaryotic cells is maintained by a well-tuned balance between biosynthesis and degradation of proteins. The ubiquitin proteasome system (UPS) constitutes the main non-lysosomal intracellular protein degradation pathway that comprises three major components: the proteasome holoenzymes, a number of ubiquitin ligases and a broad variety of deubiquitinating enzymes (DUBs). Ubiquitin (Ub) is attached to target proteins by the cascade-like catalytic action of E1, E2 and E3 enzymes. The ubiquitin molecule is activated by an E1 ligase, followed by the transfer to one of numerous conjugating enzymes, and finally an E3 ligase catalyzes the transfer of ubiquitin onto the ε-amino group of a Lysine residue within the target protein (for review see [1]). Thereby, the attachment of single Ub molecules can lead to monoubiquitination or multiple monoubiquitination, whereas binding of at least 4 ubiquitin moieties, conjugated *via* the internal Lysine 48 or 63 of ubiquitin, is called polyubiquitination. Monoubiquitination as well as Lysine 63-linked polyubiquitination regulates protein functions in processes such as DNA repair, signal transduction, endocytosis and many others. K48-linked polyubiquitination represents the canonical signal for degradation of the target protein by the 26S proteasome (for review see [2]). However, all ubiquitin modifications can be reversed by the isopeptide-bond specific proteolytic activity of DUBs [3].

More than 90 DUBs have been discovered so far and they can be divided into 5 families: cysteine proteases; ubiquitin specific protease family (USP); ubiquitin C-terminal hydrolases (UCHs); ovarian tumor proteases (OTS); the Josephine family, as well as zinc metallo-proteases JAB1-MPN-Mov34 (JAMMs) [3,4]. Generally, DUBs play different roles in almost every cellular process: They maintain the intracellular pool of free ubiquitin by removing polyubiquitin chains as well as monoubiquitin from post-translationally modified proteins in a highly specific manner, thereby regulating the stability and function of these proteins [5]. In addition to cellular DUBs, a number of virus-encoded DUBs have been identified in various virus families. It has been reported that certain viruses utilize DUB activity to evade host antiviral immune response and promote virus replication (for review see [6]). Particularly in the herpesviridae family a variety of DUBs play an important role in the virus life cycle, e.g., UL36USP of Herpes simples virus 1 (HSV-1) [7,8], human cytomegalovirus HCMV tegument protein pUL48 [9], and large tegument protein BPLF1 of Epstein-Barr virus (EBV) [10,11].

Specific DUB-inhibitors (DIs) are now available and offer the opportunity to selectively modulate DUB activities with a relatively low range of toxicity and off-target activities [12]. Some DIs have entered clinical development and have proved to be interesting candidates in tumor therapy [13,14]. Until now, DIs have mostly been considered as therapeutic drugs in oncology and inflammatory diseases [15]. The role of DUBs in infectious diseases has been only reported in rare cases: Some viruses up-regulate the activity of DUBs, which is beneficial for their replication capacity [16]. However, the potential of DIs in anti-viral therapy has not been unraveled yet, despite some episodic evidence for the inhibition of virus replication by DIs in case of Noro-, Encephalomyocarditis-, Sindbis-, Adeno- and Dengue-Virus infection [17,18,19,20]. Moreover, it was reported that the inhibition of a virally encoded DUB, the papain-like protease (PLpro), interferes with the replication of the severe acute respiratory syndrome (SARS)-Coronavirus [21].

Yet, the function of DUBs in retrovirus replication remains enigmatic. Since the original observation that retrovirus particles are enriched in free ubiquitin [22] the role of the UPS in retrovirus budding has been subject of intensive research (for review see [23,24]). Particularly the following three processes have been investigated recently:

First, the Pr55 Gag polyprotein is polyubiquitinated and the extent of polyubiquitination is somehow regulated by its late (L-) assembly domains [25,26]. It was also shown that Lysine residues 27 and 33 within the C-terminal p6 domain of human immunodeficiency virus type 1 (HIV-1) Gag are specifically monoubiquitinated [27], whereby Lysine 27 is also sumoylated [28]. The function of both modifications, however, is still uncertain. The ubiquitin acceptor sites within p6 seem to be dispensable for replication, release, and maturation of progeny virions, at least in certain host cell types [29]. Other observations indicate that monoubiquitination most likely occurs at multiple sites within all Gag domains [30,31]. Very recently, it has been shown that highly conserved motifs within the C-terminal p6 domain of Gag regulate the membrane association of Gag, accompanied by prolonged interaction with the membrane-associated CCDC8 protein complex, and thereby its entry into the UPS and major histocompatibility complex (MHC)-I pathway, independently of L-domain function [26,32,33,34].

Second, certain ubiquitin ligases are involved in virus assembly and budding. For instance, it was shown that three ubiquitin ligases regulate the transport of Gag molecules to the cell surface: Tal (Tsg101-associated ligase) [35] and Mahogunin [36], which ubiquitinate Tsg101 and thus regulate its activity, and POSH (plenty of SH3-domains) that functions in sorting of Gag to the plasma membrane *via* the trans-Golgi network [37].

Third, proteasome inhibitors not only interfere with L-domain function, necessary for efficient release of infectious HIV-1 particles, but they also cause the accumulation of polyubiquitinated Gag in cell and virions [29,38]. Since DUBs have the capacity to counteract those three processes, it was legitimate to ask of whether DIs can interfere with late processes of HIV-replication.

The HIV-1 Gag polyprotein Pr55 is necessary and sufficient for generation of virus-like particles (VLP) (for review see [39,40]). Starting with, or shortly after virus release, Gag is processed by the viral protease (PR) into its structural components: matrix (MA), capsid (CA), nucleocapsid (NC), and its C-terminal p6 protein as well as two spacer peptides (SP1 and SP2). Upon activation of PR the Gag components undergo structural rearrangements within the virion, leading to the formation of the cone-shaped core structure, which is typical for an infectious virus particle [41].

In the present study, we demonstrate that DIs, specific for USP7 and USP47, interfere with Gag processing and infectivity of released virions. In concert, these DIs reduce the replication capacity of HIV-1 in a dose-dependent manner without affecting cell viability. Most strikingly, combinatory treatment of human lymphoid aggregate cultures (HLAC) with sub-effective amounts of DIs and proteasome inhibitors revealed strong synergistic antiretroviral activity. In addition, we demonstrate that, according to expectations, DIs lead to the accumulation of polyubiquitinated Gag species and thus cause enhanced entry of Gag into the MHC-I pathway. Our cumulative data suggest that DIs might offer a novel option in antiretroviral therapy as they interfere with virus spread and simultaneously have the potential to improve immune recognition of HIV-1^+^ cells.

## 2. Materials and Methods 

### 2.1. Cell Culture and Transfection

HeLa SS6, HeLa TZM-bl, and HEK293T cells were maintained in Dulbecco’s Modified Eagle’s Medium (DMEM) containing 10% (*v*/*v*) inactivated fetal calf serum (FCS), 2 mM l-glutamine, 100 U/mL penicillin, and 100 μg/mL streptomycin. HeLa-K^b^ cells were cultivated in DMEM with additional 1 mg/mL geneticin. All cell culture media and reagents were purchased from Gibco (Life Technologies, Carlsbad, CA, USA). CEMx174 cells were cultured in RPMI Media 1640 supplemented with 10% (*v*/*v*) inactivated FCS, 2 mM l-glutamine, 100 U/mL penicillin, and 100 μg/mL streptomycin. Confluent monolayers of HeLa cells were transfected with Lipofectamine 2000 (Life Technologies) according to the manufacturer’s protocol. Twenty-four hours post transfection cells were lysed in radio immunoprecipitation assay (RIPA) buffer (150 mM NaCl, 50 mM Tris-HCl pH 8.0, 1% NP-40, 0.5% Na-deoxycholate, 0.1% sodium dodecyl sulfate (SDS), 10 mM ethylenediaminetetraacetic acid (EDTA)) containing protease inhibitor cocktail Complete (Roche, Basel, Switzerland), 5 mM *N*-ethylmaleimide (NEM), and 1 mM phenylmethylsulfonylfluoride (PMSF).

### 2.2. Expression Plasmids

The NL4-3 [42] derived expression construct pNL*env*1 [43], the R5-tropic derivative carrying the 005pf135 V3 loop region in Env [44] and the p∆R construct [30], harboring a mutational inactivation of the viral protease (PR^−^) [32], have been previously described. The expression plasmids encoding a codon optimized synthetic Gag polyprotein (syngag), which had developed from the HIV-1_HX10_ isolate [45], harboring *wt* p6 or mutants thereof, in which the two amino acid motifs PTAP have been exchanged to AIVA, have been described elsewhere [26,46,47]. The construct containing the ovalbumin-derived model epitope SIINFEKL (SL) in its p2 spacer region, syngag-SL, has been previously described [48,49]. The plasmid for expression of hemagglutinin (HA)-tagged ubiquitin (HA-Ub) was kindly provided by H.-G. Kräusslich and has been described elsewhere [30].

### 2.3. Viruses

Virus containing cell culture supernatant was harvested 48 h after transfection of HEK293T cells with proviral expression constructs and passed through a 0.45 µm pore-size filter. Virus was pelleted through 20% (*w*/*v*) sucrose (20,000× *g*, 4 °C, 90 min). Alternatively, CEMx174 cells were infected with cell culture supernatant containing HIV-1_4lig7_ [50,51]. After syncytium formation, supernatant was harvested and passed through a 0.45 μm pore-size filter. Virus stocks were normalized for p24, as quantified by p24 ELISA (Aalto Bio Reagents, Dublin, Ireland). Aliquots were stored at −80 °C.

### 2.4. Inhibitors

PR-619 and P22077 were purchased from Calbiochem (Darmstadt, Germany) and used at the concentrations indicated in the different experiments. P5091 was purchased from Selleckchem (Munich, Germany) and used at the concentrations indicated in the different experiments.

### 2.5. Cultivation and Preparation of Primary Cells

Human tonsils were received a few hours after excision, during routine tonsillectomy, from the Department of Otorhinolaryngology, Head and Neck Surgery, Friedrich-Alexander University Erlangen-Nürnberg (FAU), Germany. The cells were prepared and infected as described earlier [52,53]. HLAC were prepared by cutting the tonsils into small blocks of 2–3 mm and grinding the tissue through the sieve of a cell strainer (70 μm, BD Falcon, Bedford, MA, USA) with a syringe plunger. Cells were seeded in a 96 well plate at a concentration of 2 × 10^6^ cells per well. HLACs were cultured in RPMI Media 1640 supplemented with 15% (*v*/*v*) inactivated FCS, 2 mM l-glutamine, 100 U/mL penicillin and 100 μg/mL streptomycin, 2.5 μg/mL Fungizone, 1 mM sodium pyruvate, 1% (*v*/*v*) Eagle`s minimum essential medium (MEM) non-essential amino acid and 50 μg/mL gentamicin.

### 2.6. Ethical Statement

The study was conducted in accordance with the Declaration of Helsinki, and the protocol was approved by the Ethics Committee of the Medical Faculty of the Friedrich-Alexander University Erlangen-Nürnberg (Project identification code: 3761).

### 2.7. Investigation of Gag Processing and Virus Release by Steady State Analysis

HeLa cells were transiently transfected with pNL*env*1 *wt*. After overnight cultivation at 37 °C the cell culture supernatant was harvested and VLPs released into the supernatant were pelleted through 20% (*w*/*v*) sucrose by centrifugation (20,000× *g*, 4 °C, 90 min), washed with ice cold PBS, centrifuged (20,000× *g*, 4 °C, 90 min), lysed in SDS-polyacrylamide gel electrophoresis (PAGE) sample buffer containing 2% SDS, and boiled for 5 min. The cells were detached and washed twice in ice cold PBS and lysed in RIPA buffer, supplemented with 1 mM PMSF, 5 mM NEM, and protease inhibitor cocktail Complete (Roche). The samples were analyzed by western blotting. Protein bands were quantified using AIDA [54].

### 2.8. In Vitro Processing of Gag Polyproteins

HeLa cells were transiently transfected with p∆R PR^-^ [32]. After overnight cultivation at 37 °C, released VLPs were harvested as described above and lysed in protease cleavage buffer (50 mM MES pH 6.5, 1 M NaCl, 0.5% Triton X-100 adapted from [55]). In vitro processing was performed using 2.56 µM Recombinant HIV1 Protease Protein (Abcam, Cambridge, UK) for 9 h at 37 °C. Processing was terminated by addition of SDS-PAGE sample buffer and boiling.

### 2.9. SDS-PAGE and Western Blotting

Protein samples were separated by SDS-PAGE [56] and subsequently transferred onto polyvinylidene fluoride (PVDF) membranes (GE Healthcare, Little Chalfont, UK). Membranes were blocked with 3% bovine serum albumin and incubated with the appropriate primary antibody (Ab). Gag was detected by a rabbit Ab recognizing CA (Seramun, Heidesee, Germany). The anti-β-actin antibody was purchased from Sigma-Aldrich (St. Louis, MO, USA). The anti-mouse and anti-rabbit secondary antibodies coupled to horseradish peroxidase (HRP) were obtained from Dianova (Hamburg, Germany). HA-tagged ubiquitin was visualized using an HA-reactive monoclonal antibody (mAb) directly conjugated to HRP (Roche).

### 2.10. Detection of Ubiquitinated Gag

HeLa cells were lysed with RIPA buffer containing protease inhibitor cocktail, NEM, and PMSF. Lysates were adjusted to 1% SDS (*w*/*v*) and incubated at 95 °C for 10 min, subsequently diluted to 0.1% SDS and were then cleared by centrifugation at 20,000× *g* for 10 min. Gag content was recovered by immunoprecipitation with antibodies derived from HIV-1 patient sera pre-bound to GammaBind Plus Sepharose (GE Healthcare). For detection of ubiquitinated Gag proteins directly by western blotting, RIPA lysis buffer was supplemented with 20 μM lactacystin and 20 μM carbobenzoxyl-leucine-leucine-leucinal.

### 2.11. Flow Cytometry

For detection of H2-K^b^-bound SL-epitope, cells were stained with the allophycocyanin (APC)-conjugated 25D1.16 mAb (eBioscience, San Diego, CA, USA) diluted 1:100 in fluorescence activated cell sorting (FACS) buffer (5% (*v*/*v*) FCS, 0.02% (*v*/*v*) NaN_3_ in PBS). For intracellular Gag staining, cells were permeabilized using Cytofix/Cytoperm (BD Bioscience, San Jose, CA, USA). Gag was detected by staining with a FITC-labeled anti-CA mAb (KC57; Beckman Coulter, Brea, CA, USA) diluted 1:100 in Perm/Wash buffer (BD Bioscience). For detection of total H2-K^b^ molecules, cells were incubated with hybridoma cell culture supernatant containing the mAb B8-24-3 [57], followed by staining with secondary Alexa 647-conjugated anti-mouse Ab (Invitrogen, Waltham, MA, USA). Flow cytometry was performed on a FACSCalibur using CellQuest software (BD Bioscience). Data were analyzed by using the FACS Express V3 software (De Novo Software).

### 2.12. Single Round Infection Assay

HeLa TZM-bl indicator cells were seeded in 96-well plates in duplicates (4000 cells/well) and infected the next day with 1 ng vesicular stomatitis virus-glycoprotein (VSV-G)-pseudotyped virions standardized for p24 content by ELISA (Aalto Bio Reagents). After overnight incubation, fresh medium was added. 48 h later, cells were washed with PBS and infection was detected using the Gal-Screen chemiluminescent reporter gene assay system (Applied Biosystems, Waltham, MA, USA). β-Galactosidase activity was quantified as relative light units per second using a Synergy HT microplate reader and Gen5 Data Analysis Software (BioTek, Winooski, VT, USA).

### 2.13. Infection of HLA Cultures

For infection of HLA cultures, 2 × 10^6^ cells were incubated overnight with virus preparations equivalent to 1 ng of p24, and cell culture supernatant was collected every second or third day post infection. Where indicated, the cells were treated with different concentrations of the DIs P22077 or PR-619 as part of the medium change on the delineated days post infection (dpi).

### 2.14. Determination of the Replication Capacity

Virus replication was assessed by quantification of the virus-associated reverse transcriptase activity by [^32^P]-thymidine triphosphate (TTP) incorporation, using an oligo(dT)-poly(A) template as described elsewhere [58]. To determine the replication capacity of HIV-1 following infection with HIV-1_NL4-3_ or the field isolate 4lig7, the respective replication profiles were depicted as diagram (*y*-axis: RT activity; *x*-axis: dpi). To calculate the arithmetic mean of the values obtained from experiments performed in HLAC from different donors the area under the curve of each replication profile was calculated and defined as the corresponding replication capacity. In every experiment the replication capacity of the untreated *wt* was set to 100% and compared with the inhibitor treated samples.

### 2.15. Assessment of Cell Viability

Viability of infected and treated cells was assessed by the water-soluble tetrazolium salt (WST)-1 assay (Roche) according to the manufacturer’s instructions.

## 3. Results

### 3.1. The DUB-Inhibitors P22077 and PR-619 Impair Gag Processing without Affecting Protease Activity

**Figure 1:**
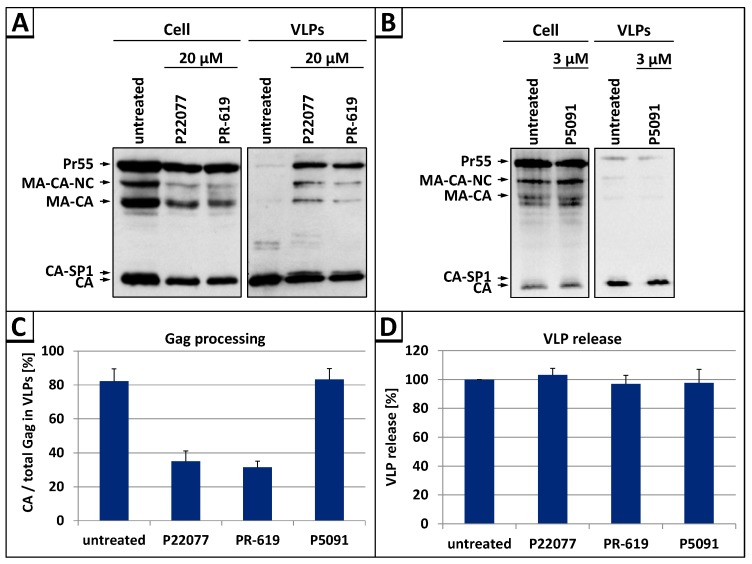
The DUB-inhibitors (DIs) P22077 and PR-619 cause a defect in Gag processing without affecting virus-like particle (VLP) release. (**A**,**B**) HeLa cells were transiently transfected with the human immunodeficiency virus type 1 (HIV-1) expression plasmid pNL*env*1. Twenty-four hours post transfection cells were treated with 20 µM P22077, 20 µM PR-619, or 3 µM P5091 for 6 h. Whole cell lysates and VLP fractions were analyzed by western blotting using an anti-capsid (CA) antibody; (**C**) Rate of Gag processing was calculated as the amount of p24 relative to the total amount of Gag in each VLP fraction. Bars represent mean values of 4 independent experiments ± SD; (**D**) The VLP release was calculated as the amount of CA in the VLP fraction relative to the total amount of Gag detected in cell and VLP fractions. Values on the *y*-axis were adjusted to 100% for the untreated control. Bars represent mean values of 4 independent experiments ± SD.

In order to investigate of whether DUB activities play any role in late steps of HIV-1 replication, we analyzed VLP release and Gag processing after DI treatment. For this purpose HeLa cells were transiently transfected with the HIV-1 expression plasmid pNL*env*1 [43] which directs efficient expression of all HIV-1 proteins except the Env glycoproteins. Cells were treated with the following DIs: P22077, which specifically inhibits the DUBs USP7 and USP47 [59], the DI P5091, which only inhibits USP7 [60], and the DI PR-619, that, in addition to USP7 and USP47, inhibits the DUBs Josephin domain containing 2 (JOSD2), deneddylase 1 (DEN1), UCH-L3, UCH-L5, USP2, 4, 5, 8, 15, 20, and 28 [59]. Western blot analyses of whole cell lysates and VLP fractions, using Gag-specific antibodies, revealed that treatment with P22077 or PR-619 led to an overall defect in Gag processing with prominent accumulation of the Pr55 Gag precursor protein as well as the processing intermediates p25 (CA-SP1), p41 (MA-CA-NC) and p39 (MA-CA) in the VLP fractions (Figure 1A). In contrast, addition of the DI P5091, which is selective for USP7, had no influence on Gag processing (Figure 1B). Densitometric analyses of the western blot data illustrate that the efficacy of Gag processing was reduced by about 2-fold in cells treated with P22077 or PR-619, whereas cells treated with the DI P5091 had *wt* level (Figure 1C), indicating that certain DUB activities regulate late steps of HIV replication. Although the DI P22077 and PR-619 clearly reduced Gag processing, none of the inhibitors had any influence on the efficiency of VLP release (Figure 1D).

**Figure 2:**
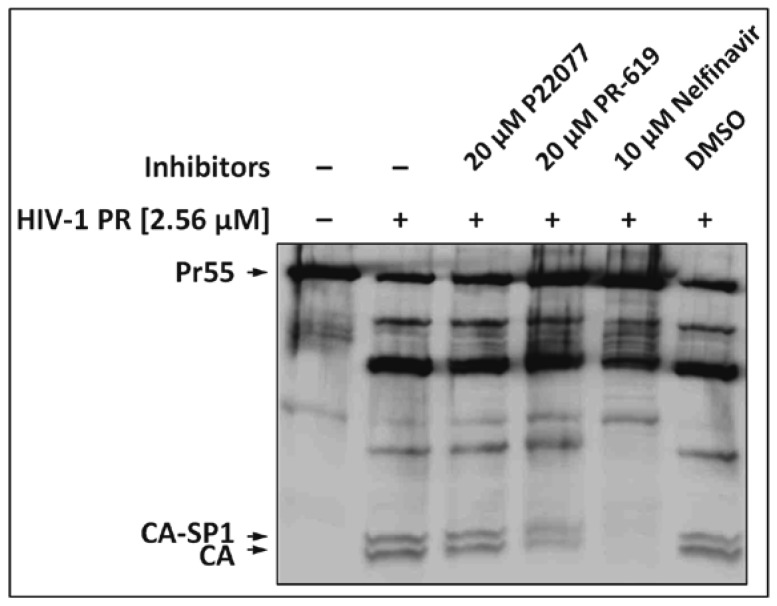
Effect of DIs on HIV-1 protease (PR) activity. HeLa cells were transiently transfected with p∆R PR^−^ expression constructs. Pr55 Gag was obtained from released VLPs and incubated for 9 h with 2.56 µM of HIV-1 PR and 20 µM P22077 or PR-619. Treatment with 10 µM Nelfinavir or dimethyl sulfoxide (DMSO) served as positive or solvent controls, respectively.

As virus release remained unaffected, one explanation for the defect in Gag processing would be direct inhibition of the HIV-1 PR by the DIs PR-619 or P22077. To test this hypothesis, we analyzed the effect of these DIs on the in vitro processing of HIV-1 Gag. Purified VLPs derived from the p∆R PR^−^ construct [32], encoding a protease deficient HIV-1 mutant virus, were lysed and incubated with 2.56 µM of the HIV-1 PR. No effect on the protease activity was observed for PR-619 or P22077 (Figure 2, lanes 3 and 4) as the efficacy of Gag processing is similar to that of the untreated control (Figure 2, lane 2), or the dimethyl sulfoxide (DMSO) solvent control (Figure 2, lane 6), respectively. The HIV protease-specific inhibitor Nelfinavir, tested in parallel, clearly blocked Gag processing (Figure 2, lane 5). Thus, the inhibitory effect of DIs P22077 and PR-619 on Gag processing cannot be explained by simply affecting PR activity.

### 3.2. The DUB-Inhibitors P22077 and PR-619 Reduce Virus Infectivity

**Figure 3:**
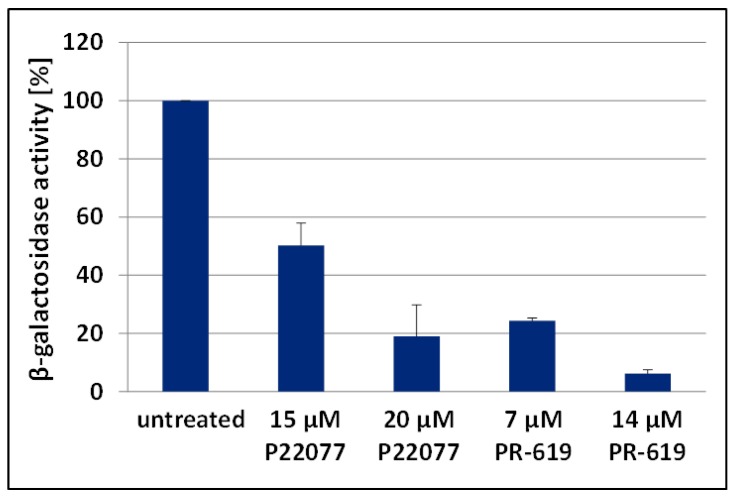
DI treatment reduces the infectivity of released virions. HEK293T cells were transfected with plasmids coding for vesicular stomatitis virus-glycoprotein (VSV-G) and pNL*env*1. The pseudotyped pNL*env*1 VLPs were collected following treatment with 15 or 20 µM P22077 and 7 or 14 µM PR-619 for 24 h. TZM-bl cells were infected with 1 ng of p24 equivalent derived from purified VSV-G pseudotyped pNL*env*1 VLPs. Infectious titers were determined by measuring the β-galactosidase activity. Bars represent the mean values of 3 independent experiments ± SD.

As treatment with P22077 or PR-619 led to reduced Gag processing, which should also reduce virus infectivity [61], we next determined the impact of these DIs on the infectivity of released HIV-1 virus particles. To perform single round infections, we generated vesicular stomatitis virus—glycoprotein (VSV-G) pseudotyped pNL*env*1 VLPs by transfecting HEK293T cells that were either incubated with the DIs P22077 or PR-619, or left untreated. VLPs were isolated and subsequently used for single round infections in TZM-bl cells, and infectivity was ascertained by measuring β-galactosidase activity. Treatment with 15 µM or 20 µM P22077 reduced the infectivity of released VLPs up to 2-fold, and 5-fold, respectively (Figure 3). Similarly, the infectivity of VLPs, generated under treatment with 7 µM or 14 µM PR-619, was about 4- or even 10-fold diminished. Thus, VLPs produced under DUB inhibition exhibit loss of specific infectivity, most likely due to impaired Gag processing.

### 3.3. The DUB-Inhibitors P22077 and PR-619 Reduce HIV-1 Replication in a Dose-Dependent Manner

**Figure 4:**
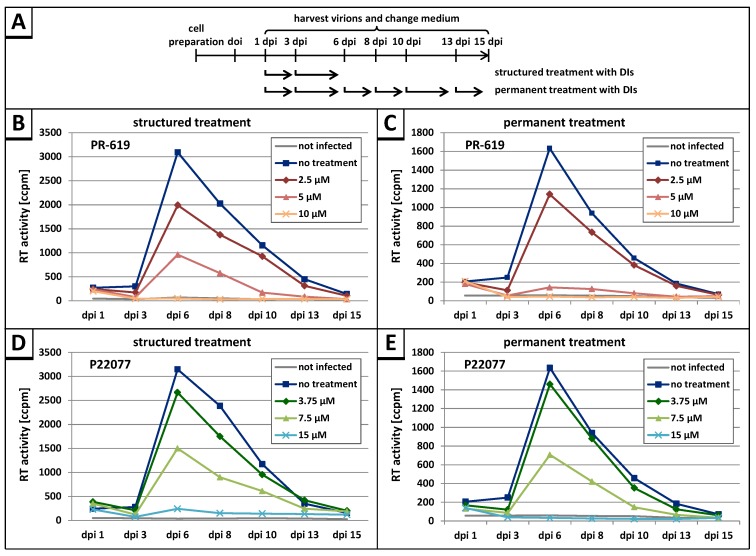
Treatment with PR-619 and P22077 reduces the replication capacity of HIV-1 in human lymphoid aggregate cultures (HLAC). (**A**) Schedule of structured and permanent treatment of HLAC with DIs; (**B**–**E**) A representative replication profile of HIV-1_NL4-3_ (X4) is shown with structured (**B**) or permanent (**C**) treatment of HLAC with 2.5 µM, 5 µM, or 10 µM PR-619 and structured (**D**) or permanent (**E**) treatment of HLAC with 3.75 µM, 7.5 µM, or 15 µM P22077 following infection with HIV-1_NL4-3_ (1ng p24). Uninfected HLAC served as negative control. Replication was assessed by quantification of the virus-associated reverse transcriptase (RT) activity contained in cell culture supernatant collected on the indicated days post infection (dpi).

To further analyze the potential antiretroviral activity of the DIs PR-619 and P22077, HLAC [62], prepared from human tonsil tissue, were infected with HIV-1_NL4-3_. HLA cultures reflect the natural cell population of a lymphoid tissue and support productive HIV-1 replication independently of coreceptor tropism and without exogenous stimulation [52,53]. The cells were infected with either T-cell (X4)- or macrophage (R5)-tropic HIV-1_NL4-3_ standardized for equal amounts of p24. Following permanent (up to 15 days post infection) or structured treatment (only day 1 and 3 post infection) with increasing concentrations of the DIs PR-619 or P22077, cell culture supernatants were collected on the indicated days post infection (dpi) and analyzed for release of virus particles by measuring the virus associated reverse transcriptase (RT) activity (Figure 4A).

Permanent treatment with the DIs PR-619 and P22077 interfered with virus replication in a dose-dependent manner, resulting in a complete loss of replication capacity at 10 µM of PR-619 or 15 µM of P22077 (Figure 4C,E). Almost the same result was observed following structured treatment, indicating a severe block in spread of infection caused by DI treatment (Figure 4B,D). No delay in peak virus replication was observed indicating the inhibitory effect of PR-619 and P22077 must set in immediately after application of the drugs.

**Figure 5:**
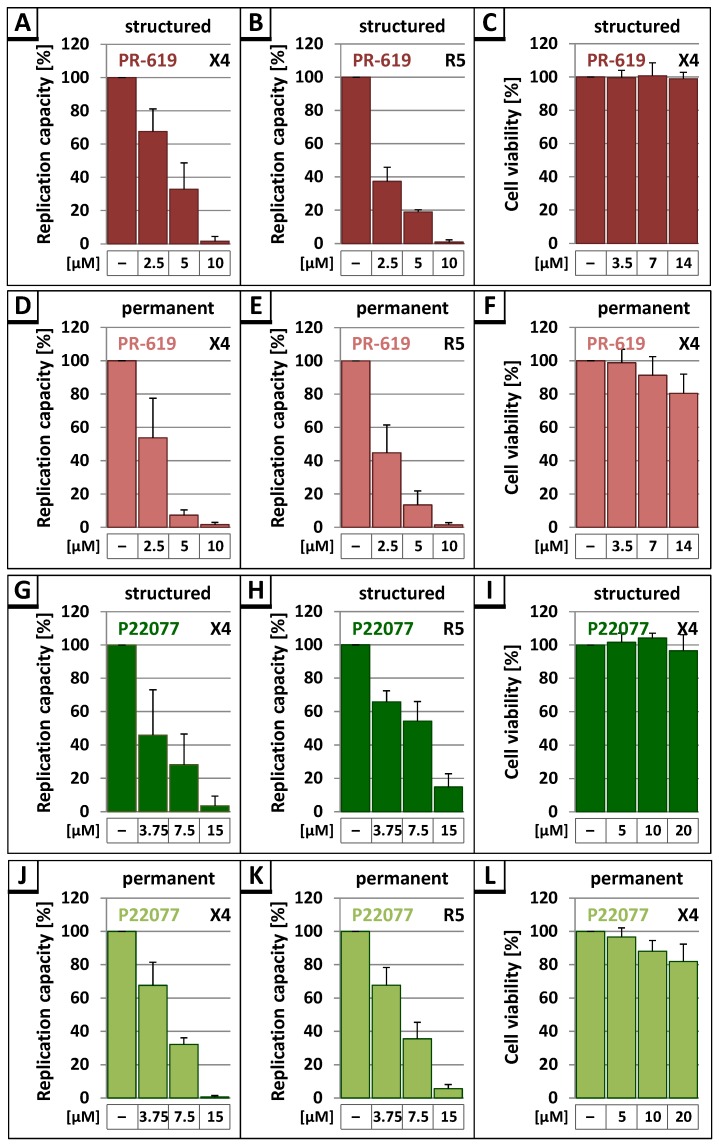
Treatment with the DIs PR-619 and P22077 leads to a concentration-dependent reduction of the replication capacity of HIV-1 in HLAC, irrespective of treatment scheme and cell tropism. (**A**,**B**,**D**,**E**,**G**,**H**,**J**,**K**) Following infection of HLAC with X4- and R5-tropic virus (1ng p24), structured and permanent treatment with 2.5 µM, 5 µM, or 10 µM PR-619 or 3.75 µM, 7.5 µM, or 15 µM P22077 respectively, was performed. The replication capacity of HIV-1_NL4-3_ was assessed by calculating the area under the curve (AUC) from each individual replication profile. The replication capacity of untreated HIV-1_NL4-3_ in each experiment was set to 100%. Bars represent the mean values of 3 (**A**,**B**,**D**,**G**,**H**,**J**) or 4 (**E**,**K**) independent experiments ± SD. (**C**,**F**,**I**,**L**) Cell viability was assessed by water-soluble tetrazolium salt (WST)-1 assay. HLAC were infected with X4- and R5-tropic virus (1ng p24), followed by structured and permanent treatment with 2.5 µM, 5 µM, or 10 µM PR-619 or 3.75 µM, 7.5 µM, or 15 µM P22077 respectively. Untreated, HIV-1_NL4-3_ infected cells were set to 100%. Bars represent the mean values of 8 independent experiments ± SD.

Since the replication of HIV-1 in HLAC generally exhibits donor-dependent differences, the area under the curve (AUC), representing the replication capacity in each HLA culture, was determined to enable the comparison of results obtained in HLAC from different donors (Figure 5A,B,D,E,G,H,J,K). Evaluation of the replication profiles of untreated *wt* compared to structured treatment with PR-619 or P22077 in HLAC revealed a concentration-dependent reduction of the replication capacity in all settings, irrespective of treatment scheme or tropism of HIV-1 (Figure 5A,B,G,H). An even more dramatic effect on X4- and R5-tropic virus replication was observed under permanent treatment with 5 µM PR-619 (Figure 5D,E) or 7.5 µM P22077 (Figure 5J,K). Irrespective of treatment schemes, the highest inhibitor concentrations of 10 µM PR-619 or 15 µM P22077 almost completely blocked the replication capacity of X4- and R5-tropic viruses. In contrast, the USP7-specific DI P5091, which had no impact on Gag processing efficiency, did not reduce virus replication (data not shown). To exclude unspecific effects of P22077 and PR-619 on cell viability, water-soluble tetrazolium salt (WST)-1 assays were performed. Structured treatment with inhibitor concentrations, which completely suppressed HIV-1 virus replication, had no impact on cell viability (Figure 5C,I). Permanent treatment merely led to a slight, stepwise decline in cell viability down to about 80% at the highest concentration of PR-619 or P22077 (Figure 5F,L). Altogether, these results demonstrate that DIs induce a concentration-dependent decline in replication capacity of HIV-1_NL4-3_, independently of tropism and treatment scheme, and without showing any toxic effects.

**Figure 6:**
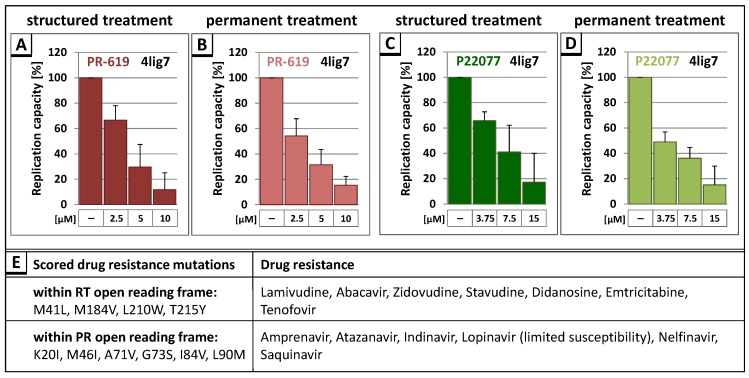
(**A**,**B**,**C**,**D**) Treatment with PR-619 and P22077 reduces the replication capacity of the multidrug resistant field isolate HIV-1_4lig7_. Following infection of HLAC with HIV-1_4lig7_ (0.5 ng p24), structured and permanent treatment with 2.5 µM, 5 µM, or 10 µM PR-619 or 3.75 µM, 7.5 µM, or 15 µM P22077 respectively, was performed. Cell culture supernatant was collected on the indicated days post infection (see Figure 4). The replication capacity of HIV-1_4lig7_ was assessed by calculating the AUC of each individual replication profile. The replication capacity of untreated HIV-1_4lig7_ in each experiment was set to 100%. Bars represent the mean values of 3 independent experiments ± SD; (**E**) Scored drug resistance mutations within the RT and the PR open reading frame and corresponding drug resistances.

Having demonstrated the anti-retroviral effect of the DIs PR-619 and P22077 for the T cell culture adapted molecular HIV-1 clone NL4-3, we examined whether those DIs also affect HIV-1 field isolates, like the multidrug resistant field isolate HIV-1_4lig7_. This isolate exhibits several drug resistance mutations within the RT and the PR open reading frame, causing resistance to all nucleoside reverse transcriptase inhibitors (NRTIs) and most PR inhibitors [63] (Figure 6E). To analyze the influence of DIs on the replication capacity of HIV-1_4lig7_, HLAC were infected with HIV-1_4lig7_ virus preparations standardized for p24, and structured or permanent treatment with indicated amounts of PR-619 and P22077 was applied (Figure 6A–D). Measuring the virus associated RT activity showed that both DIs induced the same dose-dependent reduction of replication capacity for the multidrug resistant HIV-1 field isolate, as it was observed for HIV-1_NL4-3_.

**Figure 7:**
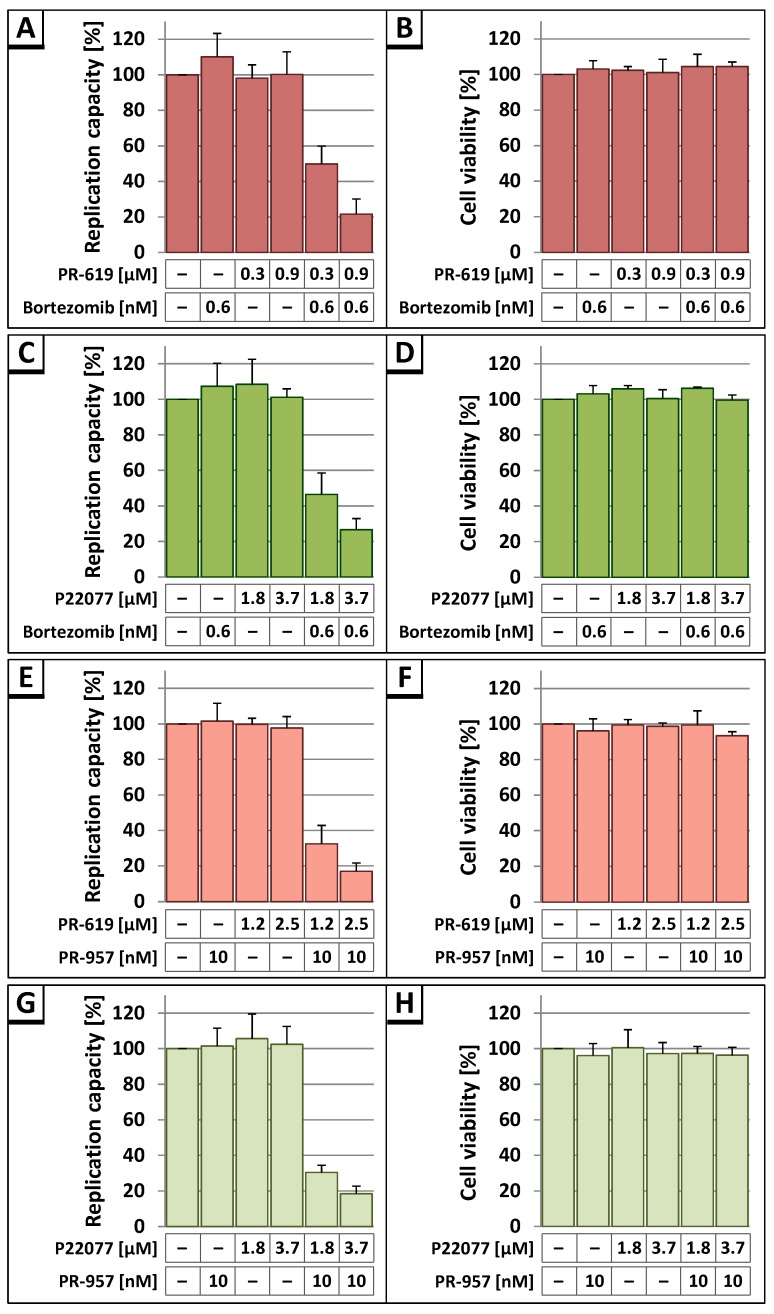
(**A**,**C**,**E**,**G**) Combinatory treatment of HLAC with DIs PR-619 or P22077 together with proteasome inhibitors Bortezomib or PR-957 leads to a reduced replication capacity of HIV-1. Following infection of HLAC with HIV-1_NL4-3_ (1 ng p24), permanent treatment with indicated amounts of PR-619 or P22077 alone, or together with 0.6 nM Bortezomib or 10 nM PR-957, respectively, was performed. The replication capacity of HIV-1_NL4-3_ was assessed by calculating the AUC from each individual replication profile. The replication capacity of untreated HIV-1_NL4-3_ in each experiment was set to 100%. Bars represent the mean values of 4 (**A**), 5 (**C**), or 3 (**E**,**G**) independent experiments ± SD; (**B**,**D**,**F**,**H**) Cell viability was assessed by WST-1 assay. HLAC were infected with HIV-1_NL4-3_ (1 ng p24), followed by permanent treatment with indicated amounts of PR-619 or P22077 alone, or together with 0.6 nM Bortezomib or 10 nM PR-957, respectively. Untreated, HIV-1_NL4-3_ infected cells were set to 100%; Bars represent the mean values of 4 (**B**,**D**) or 3 (**F**,**H**) independent experiments ± SD.

Previously, it was demonstrated that proteasome inhibitors interfere with HIV-1 Gag processing and virus infectivity [38]. Moreover, it has been shown that proteasome inhibitors block the replication capacity of HIV-1 in cell culture [64]. Both inhibitors, proteasome inhibitors and DIs, induce the accumulation of polyubiquitinated proteins, albeit by different mode of action. Thus, it was intriguing to investigate whether proteasome inhibitors in combination with DIs would generate synergistic effects on HIV-1 replication capacity. Therefore, HLAC were infected with HIV-1_NL4-3_ and replication studies were performed as described above (see Figure 4). Following permanent treatment with increasing amounts of PR-619 or P22077 together with 0.6 nM of the proteasome inhibitor Bortezomib, specific for the constitutive proteasome [65], significant reduction in replication capacity was observed (Figure 7A,C). Similar results were obtained after permanent treatment with increasing amounts of PR-619 or P22077 and 10 nM PR-957, which is a specific inhibitor of the immunoproteasome [66] (Figure 7E,G). In contrast, individual treatment with each DI or proteasome inhibitor alone had no influence on virus replication at this concentration range. Cell viability was assessed by WST-1 assay showing that all applied substances had no impact on cell viability in any individual or combinatory treatment scheme (Figure 7B,D,F,H). Together, these results reveal a synergistic antiretroviral activity of inhibitors that act on both components of the UPS, proteasomes and DUBs.

### 3.4. The DUB-inhibitors P22077 and PR-619 Cause an Enhanced Entry of Gag into the UPS and thus into the MHC-I Antigen Presentation Pathway

**Figure 8:**
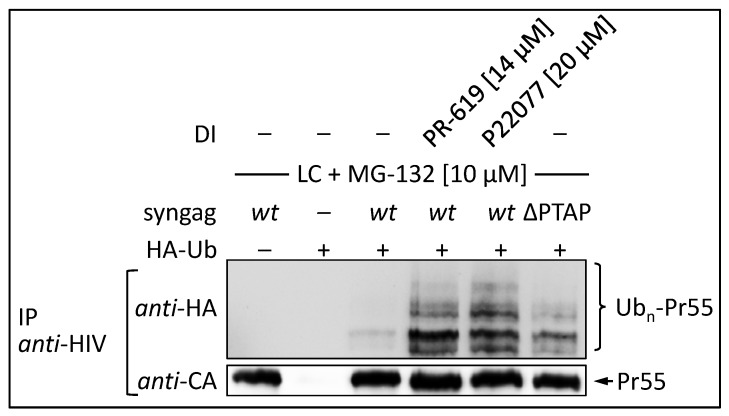
Treatment with the DIs PR-619 or P22077 leads to enhanced HIV-1 Gag ubiquitination. HeLa cells were co-transfected with HA-tagged ubiquitin and syngag *wt* or ∆PTAP expression plasmids. Twenty-four hours post transfection cells were treated with 14 µM PR-619 or 20 µM P22077 for 12 h, as well as with 10 µM LC and MG-132, 5 h before immunoprecipitation. Gag was immunoprecipitated from whole cell lysates using anti-HIV antibodies and Gag ubiquitination was detected by western blot stained for HA. The amount of precipitated Gag was detected by anti-CA staining.

By blocking the deubiquitination of Gag, the DIs P22077 and PR-619 should also increase steady state levels of poly-ubiquitinated Gag and thus its entry into the UPS. To address this issue, HeLa cells were co-transfected with HA-tagged ubiquitin (HA-Ub) and syngag expression plasmids, and treated for 12 h with indicated concentrations of DIs. The ∆PTAP mutant, which was shown to display elevated levels of ubiquitinated Gag, was assessed in parallel [25,30]. To avoid rapid proteasomal degradation of polyubiquitinated Gag species following DI treatment, cells were incubated with the proteasome inhibitors MG-132 and Lactacystin (LC) 4 h prior to cell lysis. After immunoprecipitation with Gag-specific antibodies, western blot analyses were performed and ubiquitinated Gag species were detected by anti-HA staining (Figure 8). Following co-expression of syngag *wt* and HA-Ub, a faint pattern of high molecular weight species was observed, which most likely represents polyubiquitinated Gag. Upon treatment with 14 µM PR-619 or 20 µM P22077, the intensity of the HA-Ub-signal was clearly increased and exceeded even the ubiquitination-levels of the ∆PTAP mutant. Similar amounts of precipitated Gag were confirmed by anti-CA staining. Thus, treatment with P22077 or PR-619 enhances polyubiquitination of Gag.

**Figure 9:**
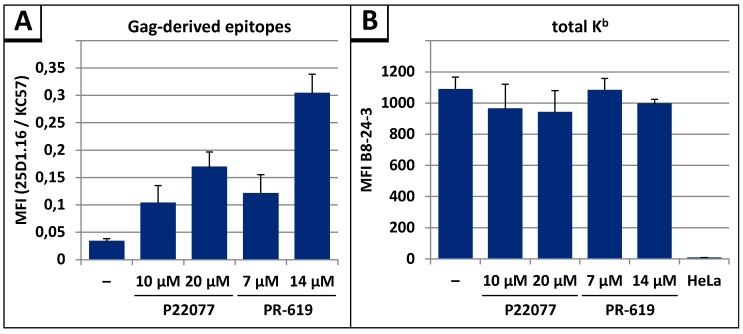
DIs P22077 and PR-619 increase antigen presentation of Gag-derived epitopes. HeLa-K^b^ cells were transiently transfected with syngag-SL expression plasmids. Twenty-four hours post transfection cells were treated with indicated amounts of P22077 or PR-619 for 4 h. (**A**) H2-K^b^-SL complexes presented on the surface of Gag-positive cells were quantified by flow cytometry using the mAb 25D1.16-APC, intracellular Gag was stained with anti-Gag Ab KC57-FITC. The mean fluorescence intensity (MFI) of the 25D1.16 staining, normalized to the MFI of the intracellular anti-Gag staining is shown. Bars represent mean values of 3 independent experiments ± SD; (**B**) Total H2-K^b^ was quantified by flow cytometry using hybridoma cell culture supernatant containing the mAb B8-24-3. HeLa cells devoid of H2-K^b^ served as negative control. Bars represent mean values of 3 independent experiments ± SD.

In our previous studies, we demonstrated that the measurement of the presentation of Gag-derived epitopes on MHC-I molecules at the cell surface represents a highly sensitive and reliable approach to follow the entry of Gag into the UPS [26,32,48,67]. To investigate whether DI treatment leads to enhanced antigen presentation, we measured the presentation of Gag-derived epitopes on MHC-I molecules at the cell surface as described previously [26,32,33,48]. After transfection of the syngag-SL expression plasmid into HeLa cells, which stably express the murine MHC-I allotype H2-K^b^ (referred to as HeLa-K^b^ cells) [68], flow cytometry analyses revealed a concentration-dependent increase in SL presentation after treatment with P22077 or PR-619 (Figure 9A). Addition of 10 µM P22077 or 7 µM PR-619 led to a 3-fold increase in SL presentation, while addition of 14 µM PR-619 caused an even 10-fold increase in the SL presentation. To exclude that the enhanced antigen presentation was due to elevated levels of H2-K^b^ complexes at the cell surface resulting from DI treatment, the amount of total H2-K^b^ was quantified, showing similar amounts of H2-K^b^ molecules at the cell surface after treatment with P22077 and PR-619 compared to untreated control (Figure 9B).

Taken together, these results indicate that DIs, especially those targeting USP7 and USP47, not only inhibit HIV-1 replication but also lead to an enhanced polyubiquitination and thus entry of Gag into the MHC-I pathway.

## 4. Discussion

It is commonly accepted that ubiquitin plays an important role in retrovirus budding, but its mode of function, particularly in HIV-1 late replication steps, has been controversially discussed [38,69,70]. Although some studies suggest that the monoubiquitination of Gag contributes to efficient virus particle release *via* the endosomal sorting complex required for transport (ESCRT) [31,71,72] more recent studies indicate that ubiquitination of Gag can occur independently of the ESCRT-complex and might be dispensable to drive viral particle release [26,32,34,73,74]. Moreover, it was demonstrated that the mutation of the PTAP-motif within p6 causes enhanced ubiquitination of Gag due to its prolonged membrane association [25,26]. This process has been ascribed to the interaction of Gag with a complex consisting of CCDC8, Obsl1, and the E3 ligase Cul7 at the plasma membrane [34]. In addition, it has been shown very recently that the highly conserved S40 and the glutamic acids within p6 can regulate the association of Gag with membranes and thus its entry into the UPS and MHC-I pathway [32,33]. Mutation of these motifs leads to a prolonged membrane association of Gag and thus an enhanced entry into the UPS. These observations led us to speculate that in the *wt* situation certain DUBs interact with p6 or other domains of Gag, remove polyubiquitin chains, and thereby regulate its entry into the UPS. In this study, we could show that DIs, especially those inhibiting USP7 and USP47, enhance the entry of Gag into the UPS and thus into the MHC-I pathway, indicating that specific DUBs play an important role in the regulation of Gag ubiquitination. Moreover, Gag processing was impaired upon DI treatment, resulting in a reduced infectivity and replication capacity of HIV-1, which can be boosted by combinatory treatment with proteasome inhibitors.

Until now, the role of the vast variety of DUBs in the replication cycle of viruses is poorly understood, although there is an increasing evidence that besides HIV also other virus families evolved strategies to hijack the UPS in order to accomplish their own replication successfully. For instance, it could be shown that Ub-ligases as well as the 26S proteasome play an important role in various steps of virus replication, e.g., entry, maturation and latent virus reactivation [75,76,77,78]. Regarding the role of DUBs in HIV-1 replication, it has been shown that the ESCRT-III interacting DUB AMSH (associated molecule with the SH3 domain of STAM) can inhibit viral particle production, while AMSH knockdown did not influence virus release [79]. Interestingly, AMSH has been reported to bind to the ESCRT-III component CHMP3 upon coexpression, which then acts as inhibitor of HIV-1 release [80]. Recently, selective DIs have become available and facilitate the investigation of the role of DUBs in viral replication. Some of these DIs allow selective targeting of just one of the 90 known DUBs which might be one explanation for their relatively low toxicity [81,82,83,84]. However, an antiretroviral activity of DIs has been reported herein for the first time.

Despite the tremendous effort that has been made to improve the classical, combined antiretroviral therapy (cART), which mainly blocks mutation-prone viral targets, there is an increasing risk for treatment failure resulting from drug resistance, incompliance, and adverse effects. While there is still a great medical need for continuous development of novel antiretroviral drugs [85], the focus is currently on cellular targets that are genetically a million times more stable and thus harbor a much lower risk of drug resistance [86,87]. Small molecules, which affect cellular targets, offer a treatment alternative for interruption of cART in case of drug intolerance or development of multi-resistant HIV-1 variants. In this study, we could provide first evidence that the DIs P22077 and PR-619 not only block the replication of the HIV-1 T cell culture adapted molecular clone NL4-3, but also of the multi-resistant field isolate HIV-1 4lig7, which possesses resistance against all NRTIs and most of the protease inhibitors used in cART [50,51]. Moreover, we could demonstrate that structural treatment with DIs P22077 and PR-619 on day 1 and 3 post infection can completely block spread of infection with similar efficacy as permanent treatment and without affecting cell viability. This indicates that a complete and comprehensive block in the production of progeny viruses, occurring early during spread of infection in the cell culture, must have been achieved by DI treatment that efficiently enables eradication of virus replication. One explanation for this observation might be the reduced infectivity of virions released under DI treatment, which is most likely due to impaired Gag processing. However, additional impact of DIs on HIV-1 genomic RNA packaging, or the incorporation of the Env glycoprotein into budding virions, as well as on the activities of regulatory proteins, like Tat, Rev or Vif, might also account for this phenomenon.

The DIs P22077 and PR-619, which share the same cellular targets, namely USP7 and USP47, but not the USP7 specific DI P5091, exhibit antiretroviral activity indicating that USP47 alone or together with USP7 might play a major role in HIV-1 replication. It has been shown previously that USP47 is specific for the cleavage of K48-linked polyubiquitin chains and thus prevents the proteasomal degradation of substrates [88]. We could show that treatment with DIs targeting USP47 leads to an enhanced polyubiquitination and entry of Gag into the MHC-I pathway, indicating that USP47 somehow interacts with Gag and prevents its entry into the UPS.

It has been shown previously that the inhibition of the proteasome leads to an accumulation of polyubiquitinated Gag species, which interfere with Gag-processing [38]. Later, it was shown that proteasome inhibitors interfere with HIV-1 replication in human peripheral blood mononuclear cells (PBMC) [64]. In this study, we could show that the addition of the DIs leads to an accumulation of polyubiquitinated Gag species and a reduction in Gag-processing. Thus, we speculated that the combinatory treatment of HLAC with DIs and proteasome inhibitors might synergistically inhibit virus replication. Si et al. have previously shown that DIs boost the inhibitory effect of proteasome inhibitors on Coxsackie virus replication [89]. However, in this case DIs alone had no effect on replication of Coxsackie virus and there was no supra-additive effect detectable, even when both inhibitors were added at high concentrations. Possibly, both inhibitors, proteasome inhibitors and DIs, are necessary to efficiently reduce the pool of free Ub, which has been shown to be necessary to block Coxsackie virus replication [89]. On the contrary, our data illustrate that DIs can enhance the effect of proteasome inhibitors on the inhibition of HIV-1 replication at very low, supra-effective concentration levels of both inhibitors. When applied individually, neither proteasome inhibitors nor DIs had any influence on the HIV-1 replication at these low concentrations. Thus, our results suggest that both inhibitors, DIs and proteasome inhibitors induce synergistic effects, without affecting cell viability.

There are mainly two possible explanations for the synergistic effect of DIs and proteasome inhibitors: First, the amount of free ubiquitin is significantly reduced by the accumulation of polyubiquitinated proteins and the lack of recycling of polyubiquitin chains. This might interfere with the function of the ESCRT-complex as early acting ESCRT factors like ESCRT-I, ESCRT-II, and ALG-2 interacting protein X (ALIX) possess ubiquitin binding domains, and thus ubiquitin may play an important role in their substrate recognition [90,91,92,93]. Second, the addition of DIs together with proteasome inhibitors might lead to the accumulation of so-called defective ribosomal products (DRiPs). DRiPs are de novo synthesized erroneous proteins, which are rapidly degraded *via* the UPS. Originally, it was shown that the HIV-1 Gag protein is a *bona fide* substrate for the DRiP-pathway [94] and proteasome inhibitors lead to an accumulation of DRiPs [38]. Combinatory treatment of cells with DIs might lead to a further enhancement of accumulated Gag-DRiPs within the cell. Thereby, those Gag-DRiPs interfere with virus release by disturbing the highly ordered assembly of Gag molecules in a prion-like manner [38].

In this study, we observed that DIs cause a general Gag processing defect. One explanation for this phenomenon would be that DIs directly inhibit the viral protease. However, this possibility could be excluded as in vitro processing assays revealed that the applied DIs, even at high concentrations, had no effect on the viral protease. Another explanation might be that polyubiquitin chains conjugated to Gag sterically disturb the highly ordered lattice of assembling Gag and thus exert a negative effect on the highly ordered Gag-assembly during virus budding, and eventually also on the auto-activation of the viral protease. Another possibility may be the direct masking of the viral protease cleavage sites by bulky polyubiquitin adducts. Accumulation of processing intermediates has been shown previously to interfere with viral infectivity in a trans-dominant negative manner [61], which is in concert with our observations that treatment with DIs P22077 and PR-619 reduces virus infectivity in a dose-dependent manner.

The K48-linked polyubiquitination of proteins is a pre-requisite for the proteasomal degradation [95]. Although the steady state amounts of Gag were not affected by the addition of DIs, the MHC-I antigen presentation as the final consequence of proteasomal degradation, was significantly enhanced. This finding is in concert with previous studies which show that an enhanced MHC-I antigen presentation did not necessarily correlate with the diminished half-life of Gag [26,48]. A possible explanation for this observation might be that just a minor fraction of Gag enters the UPS following treatment with DIs and this does not reduce the overall level of Gag that remains non-ubiquitinated. In consistency with this possibility is the notion that only a fraction of de novo synthesized proteins (approx. 5–10%) are entering the DRiP-pathway [96]. Numerous studies show that targeting an antigen for degradation by the 26S proteasome represents a powerful approach to enhance MHC class I antigen presentation and to induce an efficient cytotoxic T-cell (CTL)-response [97,98,99,100,101,102,103,104,105,106]. We now show that also DIs can cause a significantly enhanced MHC class I antigen presentation of Gag-derived epitopes. Thus, it is legitimate to speculate that DIs not only reduce viral replication but might also induce a better CTL recognition of HIV-1^+^ cells in vivo.

## 5. Conclusions

Overall, these data support a model, whereby certain DUBs play an important role in HIV-1 replication as they regulate Gag processing and thus the infectivity of released virions and simultaneously the entry of Gag into the UPS and MHC-I pathway.
